# Development of a Body-Worn Textile-Based Strain Sensor: Application to Diabetic Foot Assessment

**DOI:** 10.3390/s25072057

**Published:** 2025-03-26

**Authors:** Rory P. Turnbull, Jenny Corser, Giorgio Orlando, Prabhuraj D. Venkatraman, Irantzu Yoldi, Kathrine Bradbury, Neil D. Reeves, Peter Culmer

**Affiliations:** 1 School of Mechanical Engineering, Faculty of Engineering and Physical Sciences, University of Leeds, Leeds LS2 9JT, UK; p.r.culmer@leeds.ac.uk; 2 Centre for Clinical and Community Applications of Health Psychology, Shakleton Building, Highfield Campus, University of Southampton, Southampton SO17 1BJ, UK; j.c.corser@soton.ac.uk (J.C.); kjb1e08@soton.ac.uk (K.B.); 3 Department of Sport and Exercise Sciences, Institute of Sport, Faculty of Science and Engineering, Manchester Metropolitan University, Manchester M15 6BX, UK; g.orlando@mmu.ac.uk; 4 Manchester Fashion Institute, Faculty of Arts and Humanities, Manchester Metropolitan University, Manchester M15 6BH, UK; p.venkatraman@mmu.ac.uk; 5 School of Health Sport And Bioscience, University of East London, London E16 2RD, UK; i.yoldi@uel.ac.uk; 6 Medical School, Faculty of Health and Medicine, Lancaster University, Lancaster LA1 4YW, UK; n.d.reeves1@lancaster.ac.uk

**Keywords:** diabetic foot ulcer, shear, sensors, wearables

## Abstract

Diabetic Foot Ulcers (DFUs) are a significant health and economic burden, potentially leading to limb amputation, with a severe impact on a person’s quality of life. During active movements like gait, the monitoring of shear has been suggested as an important factor for effective prevention of DFUs. It is proposed that, in textiles, strain can be measured as a proxy for shear stress at the skin. This paper presents the conceptualisation and development of a novel strain-sensing approach that can be unobtrusively integrated within sock textiles and worn within the shoe. Working with close clinical and patient engagement, a sensor specification was identified, and 12 load-sensing approaches for the prevention of DFU were evaluated. A lead concept using a conductive adhesive was selected for further development. The method was developed using a Lycra sample, before being translated onto a knitted ‘sock’ substrate. The resultant strain sensor can be integrated within mass-produced textiles fabricated using industrial knitting machines. A case-study was used to demonstrate a proof-of-concept version of the strain sensor, which changes resistance with applied mechanical strain. A range of static and dynamic laboratory testing was used to assess the sensor’s performance, which demonstrated a resolution of 0.013 Ω across a range of 0–430 Ω and a range of interest of 0–20 Ω. In cyclic testing, the sensor exhibited a cyclic strain threshold of 6% and a sensitivity gradient of 0.3 ± 0.02, with a low dynamic drift of 0.039 to 0.045% of the total range. Overall, this work demonstrates a viable textile-based strain sensor capable of integration within worn knitted structures. It provides a promising first step towards developing a sock-based strain sensor for the prevention of DFU formation.

## 1. Introduction

Globally, 18.6 million people are affected annually by Diabetic Foot Ulcers (DFUs) [1]. In the UK, DFUs affect up to 25% of people with diabetes during their lifetime, with less than 57% of patients alive and ulcer-free 12 weeks after a diagnosis of DFU [2] and almost 60% of patients experiencing recurrence in three years [3,4]. Integrating sensors within the human–interface boundary creates a paradox known as the observer effect [5]. Attempting to measure an environment by introducing a sensor alters the environment under investigation [6]. In wearables, a synergy exists between the environment, behaviour, and measurement of interest. In the case of physical human interaction, this can potentially significantly impact the observed response. In the specific case of wearables used to assess the interface between the foot and the shoe, occupying additional internal shoe volume can reduce the available space, increasing the pressure observed. This group of patients is particularly susceptible to changes in the shoe environment. In the literature, wearable sensing technologies have been implemented that could be used to monitor DFU-forming conditions, including monitoring temperature [7], tribo-electric nano-generators [8], perspiration [9], and electrocardiograms [10], among others. However, this work primarily focuses on interaction forces, because of their influence on DFU formation.

Diabetes can cause tissue structural changes, such as damage to blood vessels in the extremities, which in turn leads to nerve damage (diabetic peripheral neuropathy) [3,11]. Tissue exhibits increased sensitivity to applied forces, resulting in accelerated tissue degradation [11,12]. Diabetic peripheral neuropathy reduces patient pain sensitivity and, therefore, the ability to feel and react to an applied force [13]. Combined with damaged blood vessels providing less oxygen, prolonged exposure to pressure has been linked to the formation of DFUs [14,15,16], due to the onset of necrosis. Over the last 20 years, work has identified a coupling between normal (pressure) and shear forces in DFU formation [17,18,19]. A recent study engaging with clinicians linked pressure-induced damage with inactivity, due to the prolonged exposure required, whereas shear relates to active conditions such as footwear fit and activity [20]. Force sensors play a critical role in DFU prevention, as demonstrated by the history of foot-based sensing systems aimed at DFU prevention.

Insoles have been implemented in the assessment of foot performance since the early 1990s, with systems such as the Tekscan F-SCAN [Norwood, MA, USA] and Novel Pedar [Munich, Germany] [21,22]. Since then, a variety of force-sensitive resistor-based approaches have been produced [23,24,25]. Key issues affecting force-sensitive resistors include drift and part-to-part repeatability. Alternative pressure mapping methods developed include multi-model arrays [26], alternative piezoelectric materials [27], capacitive sensors [28], and fibre-optic systems [29,30]. These systems measure the vertical pressure acting perpendicular to the foot’s plantar surface, negating the shear stress components acting parallel to the plantar surface.

In the literature, researchers have reported the development of insoles capable of detecting shear since the early 1980s [31,32,33]. In recent years, a variety of technologies have been implemented within insoles to measure shear, such as inductive sensor arrays [34,35], magnetic [36,37], capacitive [38], and microelectromechanical [39]. During gait, up to 1.5× body weight is placed through the foot, resulting in a challenging environment to introduce sensitive electronics. A proposed solution is the STAMPS insoles, where a cumulative measure is taken to help identify strain-prone plantar regions [19,40]. Although some systems, such as STAMPS, focus on laboratory or clinic-based measurement for diagnosis or ongoing clinical engagement to identify at-risk areas, they do not provide real-time feedback. This paper, therefore, focuses on real-time shear force measurement in daily life. The information collected by the sensor provides feedback to the wearer so that they can modify foot loading, which would otherwise lead to the development of DFUs. The form and method of feedback will not be investigated at this stage.

Outside of the measurement of diabetic patients, in recent years a variety of methods have been developed to investigate plantar shear, such as magnetic [41] and inductive [35,42,43]. So far, the critical drawback of these methods has been the form factor and the ability to place said sensors within the human interface region. A more in-depth review of sensing technologies, including preliminary replication studies, is presented in Section 2.2, which assesses methods concerning this application.

A fundamental limitation of insoles is that they are limited to use within a shoe. Although it may not be clinically recommended, users often remove their shoes in the comfort of their homes [20]. A textile-based approach with sock-integrated sensors would enable continuous tracking with users wearing the sensing technology as they wish. In addition, insoles are not compatible with every shoe style, due to the available volume within the shoe. Although some insoles are thin, they will occupy additional volume, potentially increasing dorsal foot pressure. The increased sensitivity of tissue in people with diabetes increases the importance of technologies within the human interface. Using a textile-based approach allows us to swap items already present in everyday life, avoiding additional material and minimising the interference caused by the technology within the human interface region. In some cases, people with diabetes may be required to wear custom orthotic insoles and cannot use an instrumented insole. The textile-based approach, therefore, increases the number of people who can benefit from monitoring. Although a sensorised shoe could be implemented, significant design and performance constraints and requirements exist. Patient engagement with technology will determine how likely they are to use it daily. A shoe-based system would be limited in design, reducing the individual’s ability to choose a style, a significant concern for patients [20].

A sock-based approach lends itself to the measurement of daily activities. As they relate to DFU formation, recent work linked pressure to periods of inactivity, and shear to activity [20]. Although pressure will remain a factor during motions such as gait, the dynamic nature of pressure loading during gait may not produce the conditions required for DFU formation (extended loading). Therefore, it is proposed that during activities such as gait, the monitoring of shear may prove to be a superior standalone tool for the prevention of DFU formation and will therefore be the focus of this paper. It should be noted that measurement of pressure and shear would be optimal, and this will be addressed in future work. One of the critical challenges faced in this research field is direct measurement of shear, which typically requires the parallel movement of two planes [42,44,45]. This leads to inherent design challenges with respect to system miniaturisation and robustness. Instead, the measurement of strain is proposed as a proxy for shear. In the case of the foot, it is assumed that the shear force that deforms the foot soft tissue will simultaneously generate a strain response within a textile in contact with the soft tissue. Therefore, a strain measurement can infer that a shear force has been applied. This assumption was the primary mechanism of action that was taken forward to select and evaluate sensing modalities and materials in this research.

This paper proposes a textile-based strain sensor, in which strain is taken as a proxy of the shear at the skin. Section 2 sets out a strain sensor specification for use within the physical human interface to assess the risk of DFU. Initially, available techniques were reviewed with some replications, before selecting the material to be used in sensor development. The evaluation process included preliminary tests specific to the application of this study; therefore, it was intentionally separated from the literature review. Section 3, sets out the sensor development, including manufacturing techniques and parametric assessment, to identify a design that is best suited for the application. Section 3.4, evaluates the sensor performance and demonstrates repeatability in the sensor output and manufacture. Finally, Section 4, applies the sensor to a knitted substrate, consistent with the application, and demonstrates a sensor proof of concept.

## 2. Materials and Methods

### 2.1. Scope

This research focuses on the measurement of shear forces with a smart sock for the prevention of DFU formation. Our approach is focused on developing a system capable of integration within mass-produced socks fabricated using existing industrial knitting machines. This focus led to the creation of an application-specific scope for the evaluation of potential technologies. The scope is summarised through a set of specification requirements (Table 1).

People with diabetes are at increased risk of developing peripheral arterial disease, which can inhibit blood flow and oxygen supply to the extremities [3]. Hyperglycemia can cause the degradation of the soft tissues in the extremities, making them more vulnerable to injury [11]. Where nerve ends are damaged, neuropathy can develop, removing the perception of pain [13]. The proximity of the sensing technology to the skin and any potential openings mean that the biocompatibility of the method is essential for user safety. Although sealing from the environment can be undertaken, biocompatibility at failure must be considered to ensure that no harmful materials are released, where they could affect biological systems. Due to the tissue sensitivity experienced by people with diabetes, even seams in socks can lead to DFU formation [20]. Thus, design and material choices should minimise or eliminate ridges within the sock.

Although starting at a conceptual phase, the project focused on methods ready for translation and scale-up to higher Technology Readiness Levels (TRLs) without significant additional development. Consequently, the approach focused on sensors fabricated from commercially available materials instead of materials development, bringing about potential time, cost, and regulatory constraints. The overall technology should also be scalable and low-cost per unit to facilitate adoption within socks. Through the participation of clinical and patient stakeholders, the larger project identified what a DFU prevention system should include [20]. The points discussed were collated to produce a specification Table 1.

### 2.2. Technology Evaluation

**Figure 1:**
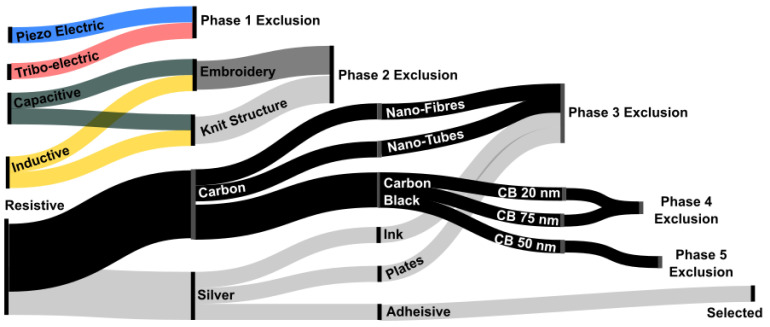
An illustration showing the routes and phases of exploration for viable sensing approaches, together with decision points on exclusion/selection. Colours are for illustrative purposes only.

During the initial investigations, the authors set out to implement or adapt preexisting strain sensor methods published in the scientific literature [33,46], reviewing them against the specification (Table 1) for appropriateness. Various technologies and approaches were investigated, covering broader electrical interaction properties such as inductive, capacitive, and resistive, followed by narrower material selection investigations such as carbon and silver-based approaches. Although optical-fibre-based sensing is an established sensing method [47], it was excluded due to the fragility of fibres, the plantar-foot’s high force environment (SID 1), and the lack of reinforcement in the textile substrate, marking optical fibres as inappropriate for the application. Magnetic approaches [48,49] were excluded, due to the requirement to include hall-effect chips, creating pressure points within the textile (SID 7). A graphical summary showing the sensor materials and methods that were explored is shown in Figure 1.

#### 2.2.1. Self-Generating

Self-generating sensor methods focused on triboelectric nanogenerators were evaluated. A fundamental mode of action is linear sliding, involving parallel anode–cathode pairs sliding against each other, which naturally lends itself to shear force detection. Several authors have demonstrated triboelectric generators in human interface applications [8,50,51,52]. Although this technology is promising for future shear sensors, production methods may not be suitable for scaled production (SID 3, 4) [51,53,54]. The ability to rapidly develop this technology and incorporate it into the selected textile was limited within the project timescales, due to the lack of compatibility with the chosen industrial knitting machine (SID 5).

#### 2.2.2. Capacitance

Capacitance-based sensors implemented through yarns were investigated. These can be implemented directly through knitting or as structures embroidered onto a textile surface. Similar efforts for other applications have investigated embroidery to produce pressure sensors in bedding [55] and chairs [56]. The possibility of implementing alternating rows within the knit structure itself was investigated. Fobelets et al. demonstrated the approach in their 2019 article [57]. The requirement of compatibility with a mass production knitting machine limited the ability to implement this method, since it severs the yarns when changing types, interrupting the continuity of the electrical pathway (SID 5). Manual reconnection of yarns would be impractical in large production volumes. Additionally, localisation (SID 6) requires the discrete distribution of sensing units across a sock, while the process set out by Foblets et al. provides a global response. Given the issues observed with capacitance-based approaches, a yarn-based inductance approach was excluded.

#### 2.2.3. Resistive: Yarns

The authors could not obtain commercially available yarns with a resistive strain response. Within the academic literature on electronic textiles, custom yarns with resistive response have been produced by layering conductive and nonconductive elements [58,59,60], surface coatings [61,62,63], and electrospinning PEDOT [64]. Although each method has demonstrated an ability to produce a resistive response under strain, they do not fit within the project specification; that is, using commercially available material, without significant or expensive processing equipment (SID 3) [62]. A preliminary investigation identified that commercially available conductive yarns would not be compatible with the required manufacturing processes, due to needle gauge specifications [65] (SID 5). Similarly, the development of a custom yarn lies outside the scope of the project and has been achieved by others in the literature [58,59,60,61,62,63,64] (SID 3). Additive manufacturing methods were identified, including silicone-encapsulated conductive materials [66] and doping methods and techniques [67,68]. Two conductive materials were selected to narrow the scope of the investigation: carbon and silver.

#### 2.2.4. Resistive: Carbon Particles

Carbon is well established within the sensor development community with variants such as nanotubes (CNTs) [69,70,71,72], nano-fibres (CNFs), carbon grease [73], carbon black powder (CB) [66,73,74], inks [75], and graphene [76] well researched within the literature. Carbon grease was not investigated, as a solid sensor was required, to mitigate the risk of leakage in the event of failure (SID 2). Although a potential solution, graphene requires high-cost equipment for its production (SID 3). Inks were not included, because future work will require washing the sensor and inks are more susceptible to washing off.

CNTs have produced a repeatable compression response [69,70,71,77]. Although an effective sensing response method, CNTs require extensive clean laboratory infrastructure as a result of their inherent toxicity. Therefore, CNTs do not meet the biocompatibility requirements, due to leaching if the sensor becomes damaged (SID 2). Furthermore, the manufacturing environment presents challenges to scalability during early development (SID 4).

**Figure 2:**
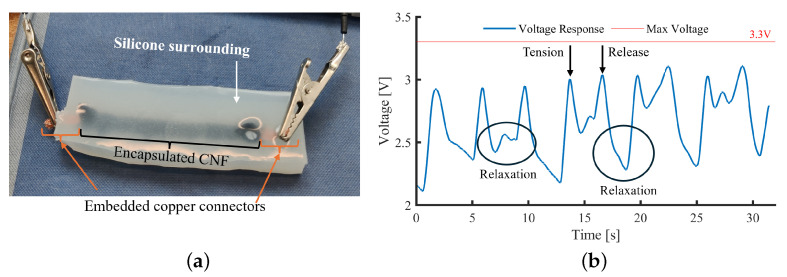
Carbon nano-fibres (CNFs) (**a**) embedded in Ecoflex 00-30 and (**b**) voltage response with relaxation regions.

CNFs were implemented, achieving conductivity; however, carbon structures are known to inhibit the curing of platinum-based silicones such as Ecoflex 00-30 [Smooth-on Inc., Macungie, PA, USA]. It was observed that achieving a mixing ratio suitable for conductivity resulted in cure inhibition. Therefore, the CNF-silicone paste was encased within silicone. A voltage strain response was observed during tension-release cycles when tested with a voltage divider setup. However, due to the non-solid nature of the paste, relaxation within the structure outside of movement was observed, reducing the voltage detected (Figure 2). While the shearing of the CNF paste could produce a response, inconsistent results are likely once a normal force is applied. Importantly, with up to 1.5× body weight put through the foot during gait, there is a significant risk of rupture (SID 1, 2).

CB has been successfully implemented in the production of stretch sensors in which the CB is set within silicone [66,73,74]. The use of CB in this application was investigated, and it is significantly safer and has a lower cost than CNTs. Attempts were made to recreate the work of Zhu et al. [66] and Muth et al. [73], who created sensors using a CB mixture embedded within a silicone substrate. CB can typically be sourced in three sizes: 20 nm, 50 nm, and 75 nm. The initial investigation used 20 nm and 75 nm CB [SGL Carbon GmbH, Wiesbaden, Germany]. It was observed that 20 nm was not sufficiently conductive; the small particles lacked electrical continuity once mixed with silicone. Conductivity could only be achieved using high ratios of CB, which inhibited silicone curing. In contrast, 75 nm produced curable silicone, but yielded high resistance (1–10 MΩ range) with poor sensitivity, making it difficult to adopt as a sensor. Therefore, the 50 nm CB used by other researchers [66,75] was selected as the middle ground regarding property response. The Wiesbaden CB was taken forward as a potential solution. The possibility of a silver-based sensor was investigated in parallel to the investigations discussed so far into yarn-based and carbon-based approaches.

#### 2.2.5. Resistive: Silver Particles

Selected for its high conductivity and antibacterial properties, silver exhibits higher conductivity when used as a sensing element. Three silver-based methods were investigated: inks, flakes, and adhesives.

Silver-conductive inks are commonly used by doping a substrate [78]. Preliminary tests found that the silver ink [Thermo Fisher Scientific, Waltham, MA, USA] saturated the knitted base layer, providing excellent conductivity but little or no detectable change in resistance with strain. Therefore, it has a poor ability to be used as a sensor. With future development, silver inks could offer an excellent method for connecting sensors and electronics along the sock’s length. However, considerations would need to be taken with respect to the washing and encapsulation of doped regions.

Another method reported in the literature involves the use of silver flakes suspended within a silicone substrate [79,80]. The silver flakes are typically coated in a lubricant for storage and dispensing, preventing the flakes from aggregating. The manufacturing process involves heating samples above 250 °C to remove most of the lubricant [81], reaching temperatures similar to the melting points of textiles such as Lycra (≈230–270 °C) and nylon (≈255 °C) commonly used in sock production. Matsuhisa et al. and Yoon et al. heated their samples to trigger the thermal decomposition of the lubricants at temperatures of 120 °C [79], 130 °C [80], and 180 °C [82]. Although conductivity was achieved, it was only achieved at 250 °C, which puts the temperature too close to the estimated melting point of nylon. The high mixing ratios required to achieve conductivity, related cost, and cure temperature meant that the use of silver flakes in their basic form fell outside the application scope (SID 4, 5).

Therefore, investigations focused on products that included silver flakes that could be cured at a lower temperature, selecting a silver adhesive [DM-SAS-10010, Dycotec Materials Ltd., Calne, UK]. Designed to replace solder in wearable technologies, the conductive adhesive provides secure connection of components and the elasticity to absorb some of the movement that would typically cause fractures in a solder-based joint, leading to delamination or reduced connection quality. In its typical application, changes in resistance would be minimal. A strain-dependent resistive response was identified, and the conductive adhesive was selected for further investigation and evaluation of its suitability for sensor production. The conductive adhesive is supplied in a syringe, which aids manufacturing through 3D printing with a fine resolution.

#### 2.2.6. Evaluation Summary

In Section 2.2, the sensing technologies were evaluated against the specification (Table 1). Two potential candidates were identified to be taken forward: 50 nm CB and conductive adhesive. Although CB options provide an effective sensing method, they are a known carcinogen and pose a risk to an already sensitive tissue boundary (SID 2). In contrast, silver has antimicrobial properties and low toxicity [83]. The conductive adhesive used in this study is commercially available and meets the higher TRL solutions and scalability requirements (SID 3, 4). Thus, conductive adhesive was selected as the basis for this work.

**Figure 3:**
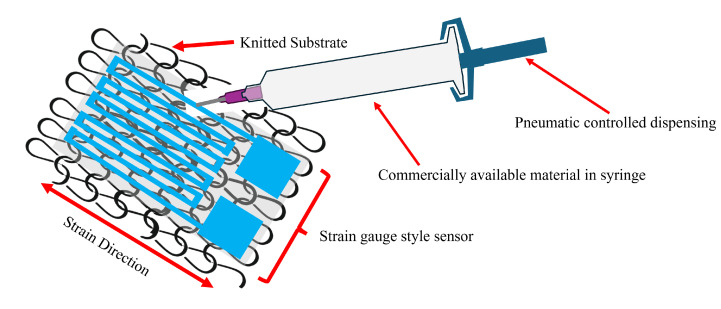
Sensor concept using commercially available silver-based conductive adhesive as the stretch element.

Tissue deformation occurs in three dimensions, with shear components representing planar aspects, normal to pressure. The sensor will adopt an established strain gauge structure, providing sensitivity of strain in a single axis of this plane (Figure 3). Although strain perpendicular to the strain gauge will induce some resistance change, the impact has been minimised through the design of the gauge geometry. Future research will develop this concept into a multi-axis array, where the effects of cross-talk between the measurement axes will be investigated further. The sensor will be manufactured through 3D printing with the supplied syringe connected to a pneumatic dispenser. A parametric design study was carried out to identify the dimensional properties of the final design.

## 3. Sensor Development

The sensor development was undertaken in three stages: (1) the production of a manufacturing platform, (2) a preliminary investigation using parametric testing to identify the sensor’s form factor, and (3) a secondary investigation focused on improving sensor robustness.

### 3.1. Design and Manufacture

**Figure 4:**
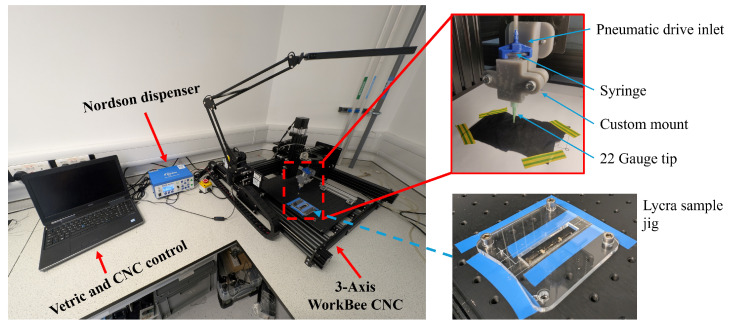
Sensing manufacturing platform integrating a WorkBee CNC with a Nordson dispenser. The red dashed box highlights the dispensing end setup, with the blue dashed line highlighting the Lycra sample jig used during sample production.

The sensor was manufactured using a 3-axis CNC linear stage with a G-code controller [WorkBee Z1+ CNC, Ooznest, Brentwood, UK], selected to provide a modular system with precise control (±precision 0.1 mm) for repeatability in sensor production. The CNC platform was augmented with a precision syringe dispenser [Ultimus V High Precision Dispenser, Nordson, Westlake, OH, USA]. The dispenser was selected for precise control and trigger system, helping automation and production repeatability. The CNC and the dispenser were connected to enable automated dispensing using a G-code trigger mechanism, as shown in Figure 4.

Although the ultimate objective of this project is to print sensors on a knitted sock fabric, a woven Lycra substrate was selected during development to speed up the development process. A laser cutter [VLS3.60DT, Denford Ltd., Brighouse, UK] was used to cut uniform 20×90 mm Lycra patch samples for printing. Each patch was secured using a laser cut jig (5 mm acrylic) onto the CNC base plate. The CNC Z axis was then zeroed on the surface of the plate using a touch probe.

The sensors were designed in CAD [Solidworks, Dassault Systèmes, France] to facilitate parametric design adaptation. Designs were exported as DXF files and imported to a G-code generator [Cut2D, Vetric, Redditch, UK] for printing. Sensors were printed by loading prefilled conductive adhesive syringes into the dispenser, fitted with a 22 gauge (0.41 mm) leur dispensing tip [Adhesive Dispensing, Milton Keynes, UK]. An iterative process identified the optimum operating parameters for printing, dispensing at 4.5 PSI, while the print head was moved at a constant 30 mm/min with a pass depth of 0.25 mm. Each sensor was made up of parallel tracks that overlapped. The sensors were cured for 30 min, with the cure temperature investigated in Section 3.2.

### 3.2. Design Parametric Testing

**Figure 5:**
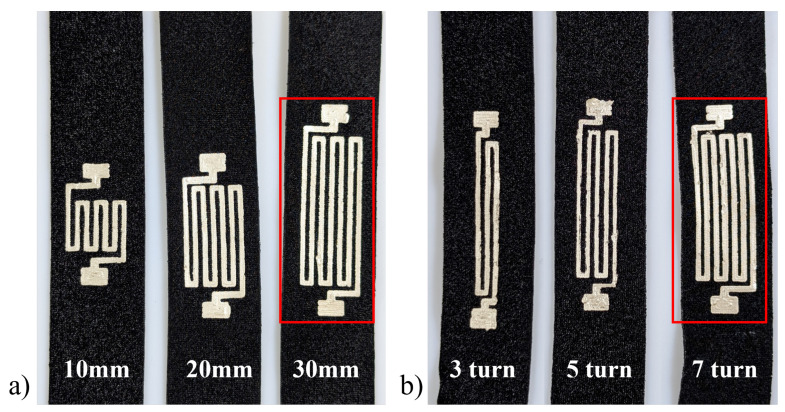
Parameters under investigation (**a**) turns and (**b**) length. The red box highlights the default sample.

The CNC manufacturing approach provided a repeatable basis for iterative design optimisation through parametric investigation. Three parameters were investigated to help improve sensor performance. First, the number of turns within the strain gauge style design was explored to enhance sensitivity to an applied strain. Three, five, and seven turns were selected, with an odd number chosen to produce polar endpoints for strain testing (Figure 5a). Secondly, the sensor length was investigated to understand the impact on performance, with potential benefits linked to an increased sensor area that encompasses more potential strain. Lengths of 10, 20, and 30 mm were selected (Figure 5). The manufacturer quotes the cure temperature of the conductive adhesive as influencing stiffness [84]. The effect on performance was investigated, and 80, 90, and 100 °C were selected as the lower end of the recommended cure range of 80–120 °C. Greater elasticity may benefit performance; therefore, a 75 °C cure temperature, below the recommended levels, was selected to assess any difference. A base setup was chosen as the default case during parameterisation, from which a single variable was altered. The sample consisted of a 30 mm long sensor with seven turns cured for 30 min at 80 °C.

All samples were prepared using the manufacturing processes described in Section 3.1 and shown in Figure 4. Three samples were produced for each parametric variation and five test repeats were taken for each sample (total *N* = 45). The preliminary testing produced dogbone samples of the conductive adhesive to perform stress-strain tests according to BS EN ISO 527 3: 2018 [85]. Due to the amount of conductive adhesive required and the cost of the material, only two samples were tested for destruction, to provide an estimate for the elastic region of the conductive adhesive. Dogbone tests provided the necessary data to identify the appropriate test parameters, determined as 10% extension as the elastic limit for 80 °C cured conductive adhesive.

**Figure 6:**
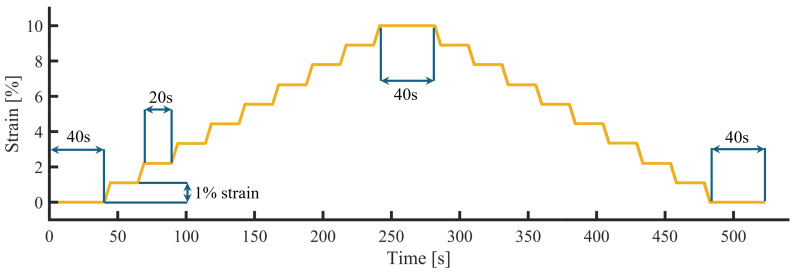
Quasi-static loading regime implemented on the single tower instron.

The testing was carried out on a single-axis universal load tester [Instron 5943, Instron, High Wycombe, UK] equipped with 250 N pneumatic jaws to secure samples safely [Instron 2712-052, Instron, High Wycombe, UK] and a 500 N load cell [Instron 2580-500N, Instron, High Wycombe, UK]. Quasi-static testing was carried out between 0 and 10% of the sensor length at intervals of 1%. Where the samples were of different lengths, this resulted in a different extension value. A 20 s pause was added between loading periods with a 40 s pause at the start, peak, and end (Figure 6).

Data were collected using a data acquisition board [MAX31865, Adafruit, New York, NY, USA] connected to a microcontroller [Arduino Mega, BCMI, Kenmore, WA, USA], which streamed data to a custom user interface [Processing IDE]. Data processing and analysis were performed in Matlab [v2024, MathWorks, Natick, MA, USA]. A data synchronisation signal marked the start and end of the load operation to align the independent datasets. A low-pass filter (f = 2 Hz) was applied to attenuate high-frequency noise from the data. Given the low speeds under investigation, a passband of two was selected. A moving average filter was applied using convolution to smooth the data using a window width of four. Following filtering, the data were normalised against the change in resistance and starting resistance to give $\Delta R/R_{0}$. Following the quasistatic loading regime, an average was taken for each window defined as the 20 s or 40 s static regions in Figure 6.

**Figure 7:**
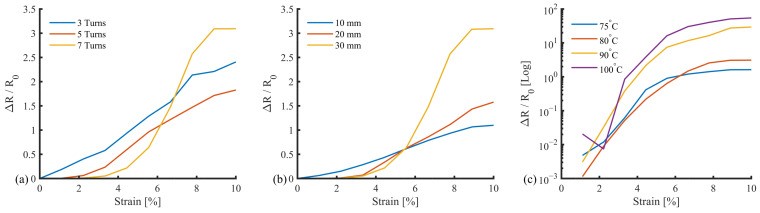
Sensor parameterisation: (**a**) Turns: three, five, and seven, (**b**) Lengths: 10 mm, 20 mm, 30 mm, and (**c**) Cure temperature 75 °C, 80 °C, 90 °C, and 100 °C.

A one-sample Kolmogorov–Smirnov test highlighted that the data were not normally distributed. A Kruskal–Wallis test was applied to determine any significant differences between datasets (Figure 7a). There was no significant difference when comparing three and five turns (*p* = 0.597), three and seven turns (*p* = 0.923), and five and seven turns (*p* = 0.827). The response curve shows that three and five turns produced a more consistent response, with standard deviations of 0.561 and 0.384, respectively. Five turns were selected, with the most consistent $\delta R/R_{0}$ response.

With respect to length, the three samples showed similar results (Figure 7b). The Kruskal–Wallis test confirmed that there were no significant differences, with *p*-values of 0.761 (10 vs. 20 mm), 0.240 (10 vs. 30 mm), and 0.634 (20 vs. 30 mm). Since the response did not vary significantly with length, a 10 mm configuration was chosen because it allows for the highest sensor density on a surface, improving localisation across an array (SID 6).

The cure temperature is known to affect the stiffness of the cured adhesive. This relationship directly influences the $\delta R/R_{0}$ response, highlighted by the log scale on the Y axis in Figure 7. Statistical analysis identified a significant difference between the 75 °C and 100 °C datasets (*p* = 0.028). The cure temperatures were paired, with a high similarity between 75 °C and 80°C (*p* = 0.986), and 90 °C and 100 °C (*p* = 0.970). Although they did not reach statistical significance, for 75 °C and 90 °C (*p* = 0.090), 80 °C and 90 °C (*p* = 0.191), and 80°C and 100°C (*p* = 0.070) there are visible differences. From these results, a curing temperature of 75 °C was selected. Together, these produced the following parameters for a base sensor design (Table 2).

### 3.3. Robustness Improvements

After parametric testing, the sensor produced a viable performance but exhibited significant variation between samples, with coefficients of variance of 0.62 ± 0.045. Consequently, two approaches were investigated to improve the robustness of the sensor: increasing the quantity of conductive adhesive, and strengthening using a secondary material. The increased quantities of conductive adhesive, while improving sensor robustness, significantly increase conductivity, reducing the sensor operating range.

**Figure 8:**
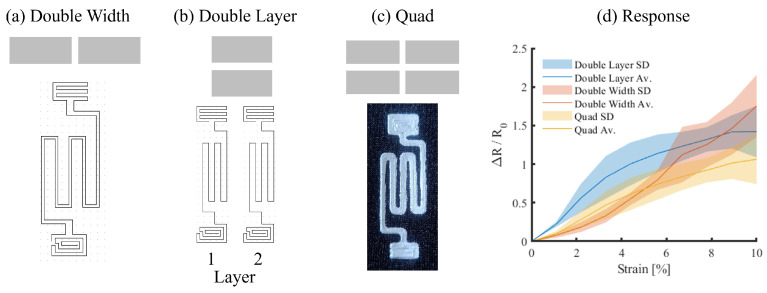
Robustness improvements: (**a**) Double Width (DW), (**b**) Double Layered (DL), (**c**) Quad and, (**d**) resistance response.

Three methods were identified to increase the quantities of the conductive adhesive present in the sensor design: double width (DW), expanding the trace width with a second pass (Figure 8a); double layered (DL), where two single-width sensor layers are stacked vertically (Figure 8b); and a combination of the two, which will be referred to as the quad setup (Figure 8c).

**Figure 9:**
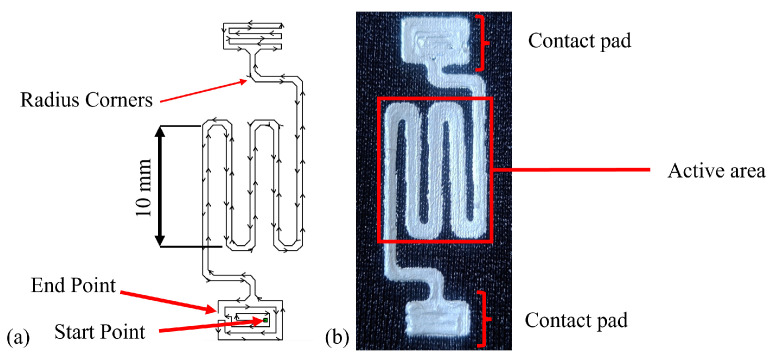
Final sensor using a quad design, (**a**) highlighting print path and (**b**) printed sensor.

The same quasi-static test method and processing described in Section 3.2 was used to assess the improvements in robustness, using a single sample tested in five repetitions for each technique (*N* = 15; Figure 8d). The addition of conductive adhesive reduced the sensitivity to strain, with $\delta R/R_{0}$ dropping to 1–1.5 compared to the peak of 2–3.5 observed during parametric tests. Although not significantly different, the selection of DW versus DL appeared to influence sensitivity at low strain levels (*p* = 0.440), although the response was lost as the strain increases. Interestingly, while there were no significant differences between DW and Quad (*p* = 0.312), a significant difference was observed between the DL and Quad samples (*p* = 0.020). As a result of additional conductive adhesive, an improvement in repeatability was observed with coefficients of variance to DL (0.222 ± 0.107), DW (0.257 ± 0.133), and Quad design (0.296 ± 0.111). The Quad (Figure 9) was selected to be taken forward, due to the superposition response of DW with a low strain response and DL with a high strain response. In the final sensor design, the corners were filleted to reduce the risk of crack propagation at turn points.

### 3.4. Lycra Performance Evaluation

Three phases of testing were used to characterise sensor performance. The first sought to investigate the influence of static drift and pressure on sensor performance. The second repeated the previously used quasistatic loading regime. Finally, sensor performance was investigated under cyclic loading conditions, representing foot loading during gait.

**Figure 10:**
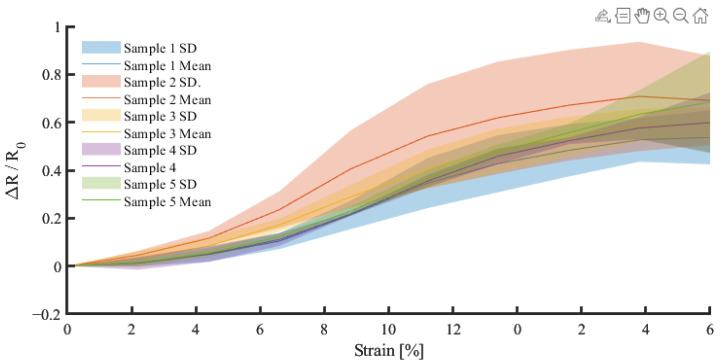
The response (dR/R) of a quad model sensor showing the characteristics of five samples.

The quasistatic test of the final quad design highlighted no significant differences between sample responses in the Kruskal–Wallis test (*p* = 0.791; Figure 10). The average percentage deviation from sample one of 11.38% demonstrates the repeatability achieved in sensor manufacture and the improved robustness of the quad form factor.

Sensors such as force-sensitive resistors exhibit resistance drift when unloaded. The sensor response was therefore investigated. Three samples were left for 10 min without applied load for the first test to identify static drift, a common occurrence in resistance-based sensors. The test was repeated five times for each sample. The sensor exhibited a gradual negative drift of 0.16 ± 0.53 Ω throughout the 10 min interval, equating to 0.037% of the total range. The observed drift was due to a relaxation effect observed in the polyurethane substrate. A similar response would be seen if a silicone-based substrate was used.

**Figure 11:**
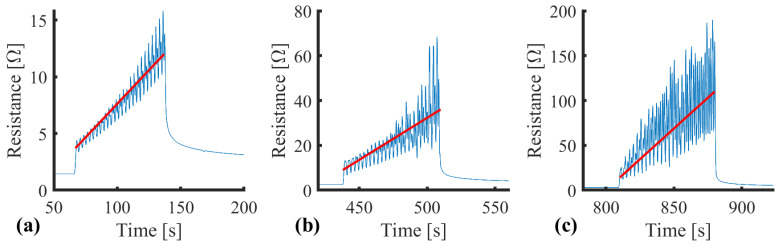
Resistance drift associated with cyclic strain. (**a**) Set 1, (**b**) Set 2, (**c**) Set 3. N.B. The continuous measurement is segmented into subplots for clarity. Red gradient for illustrative purposes.

Cyclic loading provides a representative test scenario for assessing gait and plantar loading. The sensor was evaluated over 50 cycles at 10 mm/min with three consecutive sets, representing a short walking burst. The initial investigation highlighted that a cyclic strain between 0 and 10% resulted in an upward resistance drift at increasing rates (Figure 11).

### 3.5. Mechanism of Action

**Figure 12:**
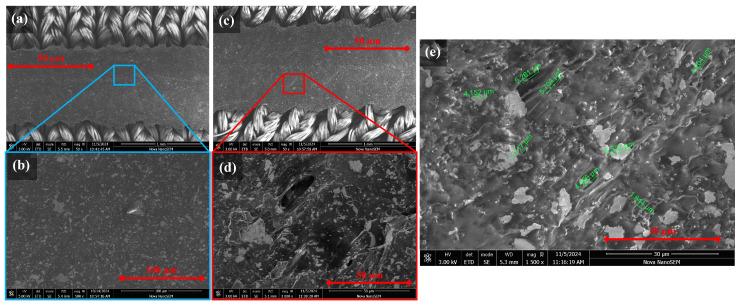
Micro-crack in relaxed and stretched silver conductive adhesive. (**a**) Relaxed 50×, (**b**) relaxed 500×, (**c**) stretched 50×, (**d**) stretched 1000×, (**e**) stretched 1500× with measurements.

Although there was a return to the baseline during unloading, when loading continued, the resistance response returned to the end point of the previous set (see Figure 11b,c), suggesting a failure-related mechanism. The mechanism of action behind the gradual upward resistance drift with dynamic movement was investigated. Given that increasing the cure temperature increased the stiffness of the conductive adhesive, it was hypothesised that this upward drift may have been related to microfractures within the elastic polyurethane substrate of the conductive adhesive. A scanning electron microscope (SEM) was used to image Lycra samples in relaxed and stretched states (Figure 12).

The SEM imaging showed a uniform surface with no evident deformation in its unstrained form (Figure 12a,b). The uniform surface aligned with the observed return to baseline resistance after removing the drift-inducing strain (Figure 11). When the sample was clamped in a position of approximately 10% strain, surface changes were visible at 50× magnification (Figure 12c). At increased magnification, there were clear strain patterns within the polyurethane substrate. Tears were observed within the substrate, creating voids (Figure 12d). These micro tears were observed down to lengths of 5.15 ± 0.24 μm, with widths of 1.68 ± 0.09 μm (Figure 12e). Similar mechanisms of action have been observed by Yoon et al. [80], who described “conductive bridges” surrounding cracks, enabling the sensor to continue performing. Similarly, Shen et al. [86] observed crack formation in their flexible sensor substrate above 10% strain.

### 3.6. Reinforcement of Sensing Element

Having identified a mechanism of action, improvements in sensor robustness were sought. This started with reassessing the cure temperature of the conductive adhesive. The manufacturer’s recommendations are between 70 °C and 120 °C. However, with a known stiffness relationship and a goal of maximising elasticity, cure temperatures between 50 °C and 80 °C were investigated at 10 °C intervals.

**Figure 13:**
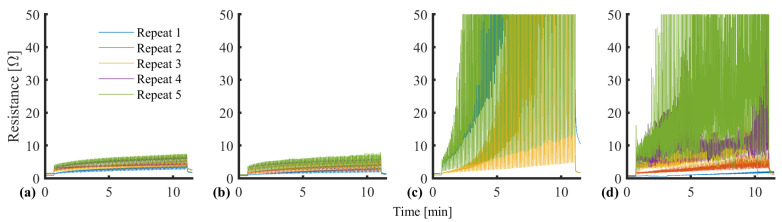
Cyclic loading response at different temperatures over 100 cycles. (**a**) 50 °C cure, (**b**) 60 °C cure, (**c**) 70 °C cure, and (**d**) 80 °C cure.

As shown in Figure 13a,b, the lower curing temperatures improved the response, significantly reducing the drift compared to baseline. This suggests that cure temperatures of 70 °C and 80 °C exhibited a response linked to microfractures (Figure 13c,d). It is expected that the manufacturer sought stiffer connections for electronics where movement is kept minimal in comparison to the dynamic nature of this application. Although there was no significant difference between 50 °C and 60 °C, the lower cure temperature was selected for the potential elastic capacity.

#### 3.6.1. Knitted Substrate Implementation

A Lycra substrate facilitated the rapid development of prototypes and methods by enabling production of uniform laser-cut samples for controlled testing. The target application requires the implementation of the sensing technology on a sock. Therefore, the method developed on a 100% Lycra sample was translated to a knitted substrate produced using an industrial sock knitting machine. The knitted sock substrate was comprised of three yarns standard in sock production; cotton for comfort and moisture absorption, Lycra for elasticity, and nylon for wash durability and strength. The selected nylon is produced from recycled car tyres.

**Figure 14:**
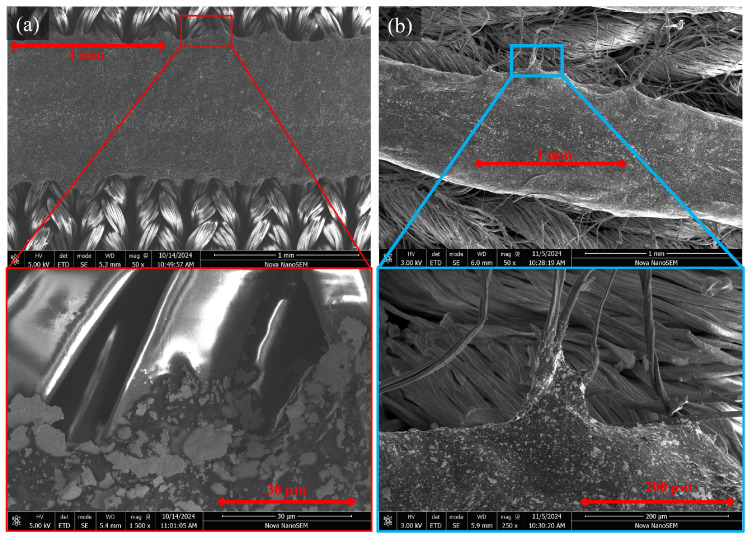
Silver conductive adhesive integration with (**a**) Lycra and, (**b**) knitted natural fibres, with highlighted interfacing.

Sensor samples were produced using the same methods as for Lycra, including the dispensing rate and the movement speed (Section 3). Although there are differences in surface properties, the movement range under investigation means that the primary influence on the force–elongation response is the individual Lycra fibres. SEM was used to investigate how the conductive adhesive integrated within the different substrate surface structures (Figure 14). The results showed structural differences between the fine woven Lycra and courser knitted multi-yarn substrate.

Figure 14a highlights the smooth and uniform nature of the Lycra yarn and the low interstitial spaces between the filaments, which prevent the formation of mechanical interlocks, resulting in low adhesion of the conductive adhesive, as highlighted by the minimal coating found along the edge in 1500× magnification (Figure 14c). In contrast, the knit yarns had protruding fibres that provided interstices distributed throughout the textile surface (Figure 14b). The stray fibres and visible surface roughness resulted in improved integration. Fibres act to reinforce the conductive adhesive to form a composite. Similarly, where contact is made between the natural fibres and the conductive adhesive, there is a higher level of ingress along the fibres (Figure 14d). During preliminary sample testing, the natural fibres formed a composite structure with the conductive adhesive, reinforcing the knitted yarns. The need to strain the sample to 20% to achieve the same resistive response observed within the 0–10% range in the Lycra samples was observed. Furthermore, a reduction in drift was observed; it is hypothesised that the composite structure reduced the frequency of microtears, in addition to the improved adherence observed in Figure 14d, improving the overall robustness.

#### 3.6.2. Sensor Encapsulation in Silicone

During the design process, the authors considered how the sensor (and sock) would interact with its environment under its intended working conditions. The primary need identified during use was protection from outside elements, such as sweat or high levels of humidity, which could form conductive paths and impede the sensor behaviour. For system longevity and hygiene, the system must undergo routine maintenance, namely washing cycles. The cured sensors were encapsulated within silicone to address these issues by providing a protective moisture-impermeable barrier. Adding silicone to the fibres inhibits the natural wicking effect of cotton, which would increase the sensor’s local humidity. In addition, any impact of moisture on mechanical properties is thereby eliminated. Washing would need to occur at a low temperature (≈30°) to ensure that sensor stiffness remained unaffected, with air drying recommended. Although the impact on the performance of clean–dry cycles was not addressed in this paper, future work will seek to identify the impact on the wearable device to ensure a device that is fit for purpose. In addition to protection, the encapsulating silicone can be selected to fine-tune the force response of the strain sensor (by selecting softer or stiffer silicones). A range of biocompatible commercial silicones were selected for investigation (Exoflex 00-20, 00-30, and 00-50).

**Figure 15:**
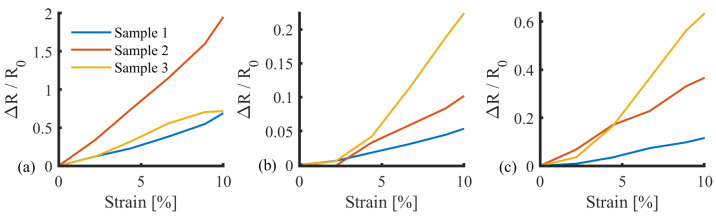
Sensor response with reinforcing silicones Exoflex (**a**) 00-20, (**b**) 00-30, and (**c**) 00-50.

The silicones reinforcing the sensor resulted in a statistically significant difference (*p*<0.05) in resistance response (Figure 15). The 00-20 silicone exhibited the lowest stiffness and provided the least support for the reported large resistances. The 00-50 silicone responded within a range similar to 00-30. However, 00-30 silicone was selected due to a more consistent response trajectory between samples, with an inter-sample standard deviation of 0.139, compared to 0.299 and 3.95 for 00-50 and 00-20, respectively.

## 4. Final Sensor Design and Evaluation

This section evaluates the performance of the finalised sensor, following the development reported in Section 3.

**Figure 16:**
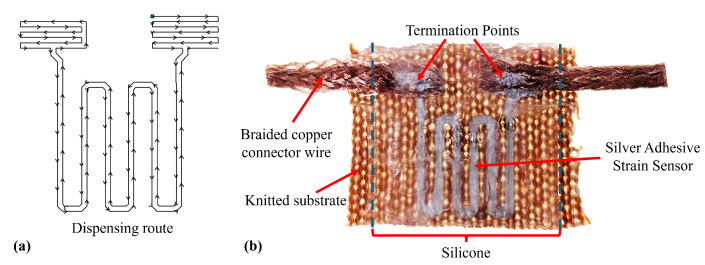
Final sensor design, showing (**a**) dispensing route, and (**b**) sensor sample.

**Figure 17:**
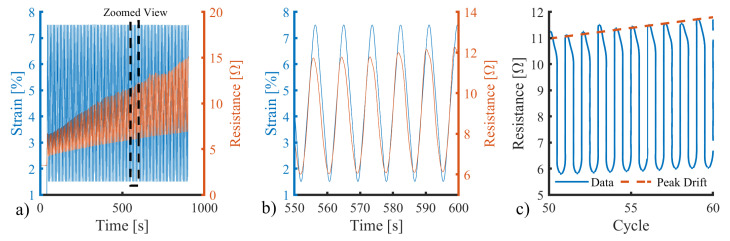
Applied strain vs. sensor response example data (**a**) over 100 cycles (left) with (**b**) zoomed-in section across six cycles highlighting conformity and (**c**) example of quantifying the dynamic peak drift present. Blue shows strain (left y axis) and orange shows resistance (right y axis).

The finalised design incorporates six turns, adding an additional turn relative to earlier prototypes, to achieve the level termination typical of the conventional strain gauge design, as shown in Figure 16. The sensor follows the “Quad” design with a length of 10 mm. The sensor was printed on a knitted substrate (Section 3.6.1) and reinforced with an Ecoflex 00-30 encapsulating layer. The test was carried out using a cyclic load regime in 100 cycles, which was repeated three times for each of the three samples using the same setup as in Section 3.2. The sample was clamped within pneumatic jaws at a distance of 10 mm, allowing the sensor to be located entirely between the jaws. This baseline start position was equal to a 0% strain. The strain was applied to the sensor at a rate of 10 mm/min, with strain moving between 1.5% and 7.5%. The prestrain was added to represent the slight stretch present when wearing a textile garment. Data analysis extracted sensitivity, dynamic drift, and hysteresis metrics to assess sensor performance. An example dataset can be seen in Figure 17, where the left axis (blue) represents the cyclic strain loading regime and the right axis (orange) represents the resistance measured across the sensor.

Although significantly reduced following sensor development, some drift remained in the sensor response, evident as an increase in the peak value throughout the 100 cycles (Figure 17a). For this study, drift was defined as the increase in resistance per cycle relative to the total measurable range (430 Ω). The three samples exhibited a dynamic drift of 0.039 ± 0.014%, 0.045 ± 0.021%, and 0.045 ± 0.019%, respectively. Relative to the measurable range of the chip (0–428 Ω), the sensor produced an output within the operating range of the data acquisition system for approximately 2275 (±562) cycles before the chip reached saturation.

**Figure 18:**
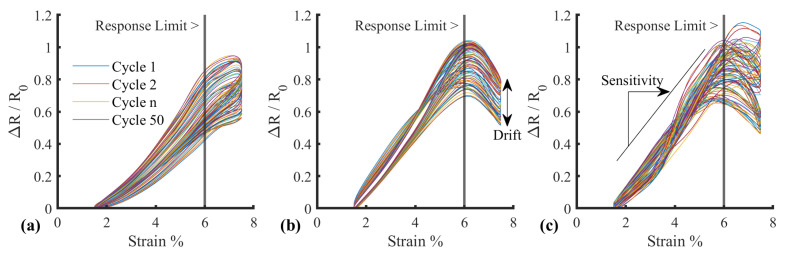
Cylic response with matched strain and normalised resistance for (**a**) Sample 1, (**b**) Sample 2, and (**c**) Sample 3. Highlighting sensitivity and drift metrics.

The final sensor, including encapsulation, operated with a reliable linear response in the range of 0–6% (Figure 17). Although lower than the response exhibited in preliminary testing, this is sufficient for the requirements of this application. The sensor had a higher stiffness than the surrounding sock, due to the silicone reinforcement. Therefore, during donning, the sock will stretch to fit the user’s foot before the sensor, ensuring the sensor is not over-strained. In addition, the 10% strain range permits a significant expansion (within the range expected during sock donning). During use (e.g., when donned), the combination of plantar pressure and silicone will ensure the sensor moves with the underlying plantar soft tissues. Based on this strain limit, the sensor sensitivity was calculated with the linear best fit gradient of strain between 1.5% and 5%, where the response is linear (Figure 18c).

**Figure 19:**
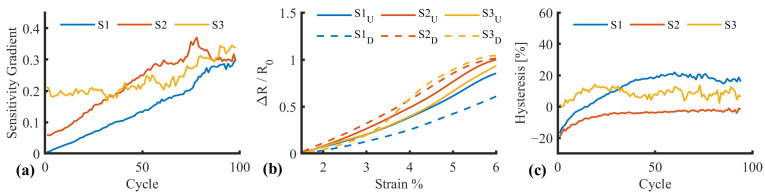
Average sensor response metrics (**a**) Sensitivity gradient progression across 100 cycles, (**b**) hysteresis loop example, and (**c**) hysteresis percentage progression across 100 cycles.

An increase in sensitivity was observed in each of the three samples during the 100-cycle test regime (Figure 19a), in keeping with the drift characteristics and the mechanism of action discussed in Section 3.5. In general, the responses followed similar trajectories, converging at a sensitivity of approximately 0.3. However, there were significant differences between the average cycles between samples one and two, and one and three (*p* < 0.05), with no significant difference between samples two and three (*p* = 0.607), highlighting the need for sensor-specific calibration.

Hysteresis was calculated using trapezoidal numerical integration [trapz, Matlab v2024b, MathWorks, Natick, MA, USA] of the response to determine the area within the hysteresis loop (Figure 19b). The output was normalised against the upward loop to represent hysteresis as a percentage (Figure 19c). In general, the response followed a gradual increase, followed by a plateau. The plateau occurred within the first 25–50 cycles, suggesting a “bedding-in” process, consistent with the related drift and sensitivity changes that manifest due to microfractures occurring over time.

## 5. Discussion

Developing strain sensors that convert mechanical strain into a measurable response is a promising solution for detecting a key causative component of DFU formation. Implementing a printed sensor using a conductive adhesive provides a practical solution for this application. A key novelty is the ability to apply the conductive adhesive repeatedly to a knitted substrate. Integrating strain-sensing technology within a knitted garment, such as a sock, provides the potential for an unobtrusive monitoring method appropriate to this sensitive environment. The evaluation of the proof-of-concept sensor highlighted conductive adhesive as a promising sensing material. Although some dynamic drift was observed under cyclic loading, this is common in flexible stretch sensors. Similar performance and characteristics were reported in other textile-based sensors. For example, Li et al. [87] observed a gradual increase in $R/R_{0}$ over 500 cycle test regimes. Similarly, Yoon et al. [80] used sensors based on silver flakes and nanoparticles encapsulated within silicone rubber and experienced dynamic drift during cyclic testing, due to microfractures or “conductive bridges”.

Reflecting on the specifications identified in collaboration with the patient and clinical engagement (Section 3) and set out in Table 1, the soft sensor developed meets the specification criteria. The sensor does not exhibit fracture under load, with silicone reinforcement for added protection (SID 1). The silicone enclosure is safe for skin contact and silver is naturally anti-microbial in the case of failure (SID 2). During development, commercially available materials were prioritised for investigation, selecting a commercially available conductive adhesive (SID 3). Although the 3D printing method may not be suitable for conductive adhesive mass production, other processes, such as screen printing, could be implemented. Therefore, the conductive adhesive has sufficient scope for mass production processes with further development (SID 4). The integration of conductive adhesive was successfully demonstrated within a knitted substrate produced on the industrial knitting machine (SID 5); future work will investigate the integration of electronics and data acquisition. The 3D printing and parametric design choices produced a sensor small enough to make an array with future development, allowing strain localisation (SID 6). Finally, with 0.25 mm print layers, the sensor has a low profile, with the ridges created by the strain gauge design filled with silicone, to provide a continuous surface (SID 7).

It should be noted that there were some limitations due to unknown factors in the application, such as the expected strain. The sensor was identified to work repeatably and reliably up to 6% deformation during dynamic movement. Future work will evaluate how this relates to plantar strain characteristics, which are currently poorly reported and understood in the literature. This sensing approach has the potential to help better understand plantar strain regimes in healthy and diabetic foot contexts and thus advance technology for instrumentation, to aid in the prevention of DFU formation.

## 6. Conclusions

In the first half, this article presented the conceptualisation and development of a strain sensor for use in e-textiles and DFU prevention. Given the application, the limited number of appropriate sensing methodologies provided a focus and guided the research. The development highlighted the good repeatability of the single sensor, with no significant differences between repeat tests. The sensor has an estimated life span of 2275 (±562) cycles. The sensor proposed in this paper provides a promising first step toward a new wearable system for the prevention of DFUs, which meets the specification set out in this work (Table 1). With further development, this technology has the potential to significantly impact patient quality of life while reducing healthcare sector expenditure. Outside of healthcare, the technology offers potential benefits to other fields, including but not limited to general shoe fit, informing accessories with shearing such as backpack straps, and sports performance monitoring.

## Figures and Tables

**Table 1:** Table of specification requirements defined by the application and scope. SID = Specification IDentifier.

SID	Name	Requirement
1	Robustness	Placed on the foot’s plantar surface up to 1.5×, bodyweight can be exerted during gait, requiring a robust solution.
2	Biocompatibility	Solutions must be biocompatible due to the increased risk of infection due to cracked or fissured skin increasing access through natural barriers.
3	Technology Readiness Level	With the interest of producing a real-world impact on patient quality of life. Solutions using commercially available technology will be prioritised.
4	Production Scale	The processes used in production must involve scalable technology.
5	Production Compatible	Relating to ID4 solutions must be compatible with industrial knitting machines.
6	Sensor Localisation	A discrete rather than global sensing approach for response localisation.
7	Low Profile	Due to increased sensitivity to ulcer formation of soft tissue, ridges and point forces must be avoided.

**Table 2:** Parameters selected to be taken forward.

Parameter	Selected Value
Number of Turns	5
Length	10 mm
Cure Temperature	75 °C

## Data Availability

The original data presented in the study are openly available at https://doi.org/10.5518/1649.

## Abbreviations

The following abbreviations are used in this manuscript:

CB	Carbon Black
CNF	Carbon Nano-Fibres
CNT	Carbon Nano-Tubes
DFU	Diabetic Foot Ulcer
DL	Double Layer
DW	Double Width
TRL	Technology Readiness Level

## References

[1] Armstrong D.G., Tan T.W., Boulton A.J.M., Bus S.A. (2023). Diabetic Foot Ulcers: A Review. JAMA.

[2] NDFC (2023). National Diabetes Foot Care Audit 2018 to 2023. https://digital.nhs.uk/data-and-information/publications/statistical/national-diabetes-footcare-audit/2018-2023.

[3] Tresierra-Ayala M.A., García Rojas A. (2017). Association between Peripheral Arterial Disease and Diabetic Foot Ulcers in Patients with Diabetes Mellitus Type 2. Med. Univ..

[4] Dubský M., Jirkovská A., Bem R., Fejfarová V., Skibová J., Schaper N.C., Lipsky B.A. (2013). Risk Factors for Recurrence of Diabetic Foot Ulcers: Prospective Follow-up Analysis in the Eurodiale Subgroup. Int. Wound J..

[5] Dirac P. (1981). The Principles of Quantum Mechanics.

[6] Baclawski K. The Observer Effect. Proceedings of the 2018 IEEE Conference on Cognitive and Computational Aspects of Situation Management (CogSIMA).

[7] Reyzelman A.M., Koelewyn K., Murphy M., Shen X., Yu E., Pillai R., Fu J., Scholten H.J., Ma R. (2018). Continuous Temperature-Monitoring Socks for Home Use in Patients With Diabetes: Observational Study. J. Med. Internet Res..

[8] Huang T., Wang C., Yu H., Wang H., Zhang Q., Zhu M. (2015). Human Walking-Driven Wearable All-Fiber Triboelectric Nanogenerator Containing Electrospun Polyvinylidene Fluoride Piezoelectric Nanofibers. Nano Energy.

[9] Jia J., Xu C., Pan S., Xia S., Wei P., Noh H.Y., Zhang P., Jiang X. (2018). Conductive Thread-Based Textile Sensor for Continuous Perspiration Level Monitoring. Sensors.

[10] Le K., Narayana H., Servati A., Bahi A., Soltanian S., Servati P., Ko F.K. (2022). Electronic Textiles for Electrocardiogram Monitoring: A Review on the Structure–Property and Performance Evaluation from Fiber to Fabric. Text. Res. J..

[11] White S., McCullough M.B.A., Akangah P.M. (2021). The Structural Effects of Diabetes on Soft Tissues: A Systematic Review. Crit. Rev. Biomed. Eng..

[12] Zhou J., Xu B., Chen W. (2015). Influence of Hallux Valgus Deformity on Forefoot Pressure Distribution of Chinese Diabetic Patients. Int. J. Diabetes Dev. Ctries..

[13] Hicks C.W., Selvin E. (2019). Epidemiology of Peripheral Neuropathy and Lower Extremity Disease in Diabetes. Curr. Diabetes Rep..

[14] Veves A., Murray H.J., Young M.J., Boulton A.J.M. (1992). The Risk of Foot Ulceration in Diabetic Patients with High Foot Pressure: A Prospective Study. Diabetologia.

[15] Chatwin K.E., Abbott C.A., Boulton A.J., Bowling F.L., Reeves N.D. (2019). The Role of Foot Pressure Measurement in the Prediction and Prevention of Diabetic Foot Ulceration—A Comprehensive Review. Diabetes/Metab. Res. Rev..

[16] Luger E., Nissan M., Karpf A., Steinberg E., Dekel S. (2001). Dynamic Pressures on the Diabetic Foot. Foot Ankle Int..

[17] Perry J.E., Hall J.O., Davis B.L. (2002). Simultaneous Measurement of Plantar Pressure and Shear Forces in Diabetic Individuals. Gait Posture.

[18] Jones A.D., De Siqueira J., Nixon J.E., Siddle H.J., Culmer P.R., Russell D.A. (2022). Plantar Shear Stress in the Diabetic Foot: A Systematic Review and Meta-analysis. Diabet. Med..

[19] Jones A.D., Crossland S.R., Nixon J.E., Siddle H.J., Russell D.A., Culmer P.R. (2023). STrain Analysis and Mapping of the Plantar Surface (STAMPS)—A Novel Technique of Plantar Load Analysis during Gait. Proc. Inst. Mech. Eng. Part H J. Eng. Med..

[20] Corser J., Yoldi I., Reeves N.D., Culmer P., Venkatraman P.D., Orlando G., Turnbull R.P., Boakes P., Woodin E., Lightup R. (2025). Developing a Smart Sensing Sock to Prevent Diabetic Foot Ulcers: Qualitative Focus Group and Interview Study. J. Particip. Med..

[21] Pitei D.L., Ison K., Edmonds M.E., Lord M. (1996). Time-Dependent Behaviour of a Force-Sensitive Resistor Plantar Pressure Measurement Insole. Proc. Inst. Mech. Eng. Part H J. Eng. Med..

[22] Hsiao H., Guan J., Weatherly M. (2002). Accuracy and Precision of Two In-Shoe Pressure Measurement Systems. Ergonomics.

[23] Chen B., Ma H., Qin L.Y., Guan X., Chan K.M., Law S.W., Qin L., Liao W.H. Design of a Lower Extremity Exoskeleton for Motion Assistance in Paralyzed Individuals. Proceedings of the 2015 IEEE International Conference on Robotics and Biomimetics, IEEE-ROBIO 2015.

[24] Anas M.N. (2014). An Instrumented Insole System for Gait Monitoring and Analysis. Int. J. Online Biomed. Eng. (iJOE).

[25] Dabiri F., Vahdatpour A., Noshadi H., Hagopian H., Sarrafzadeh M. Electronic Orthotics Shoe: Preventing Ulceration in Diabetic Patients. Proceedings of the 2008 30th Annual International Conference of the IEEE Engineering in Medicine and Biology Society.

[26] McKnight M., Tabor J., Agcayazi T., Fleming A., Ghosh T.K., Huang H., Bozkurt A. (2020). Fully Textile Insole Seam-Line for Multimodal Sensor Mapping. IEEE Sens. J..

[27] Tan Y., Ivanov K., Mei Z., Li H., Li H., Lubich L., Wang C., Wang L. (2021). A Soft Wearable and Fully-Textile Piezoresistive Sensor for Plantar Pressure Capturing. Micromachines.

[28] Aqueveque P., Osorio R., Pastene F., Saavedra F., Pino E. Capacitive Sensors Array for Plantar Pressure Measurement Insole Fabricated with Flexible PCB. Proceedings of the 2018 40th Annual International Conference of the IEEE Engineering in Medicine and Biology Society (EMBC).

[29] Guignier C., Camillieri B., Schmid M., Rossi R.M., Bueno M.A. (2019). E-Knitted Textile with Polymer Optical Fibers for Friction and Pressure Monitoring in Socks. Sensors.

[30] Leal-Junior A.G., Frizera A., Avellar L.M., Marques C., Pontes M.J. (2018). Polymer Optical Fiber for In-Shoe Monitoring of Ground Reaction Forces During the Gait. IEEE Sens. J..

[31] Pollard J.P., Le Quesne L.P. (1983). Method of Healing Diabetic Forefoot Ulcers. Br. Med. J..

[32] Wang L., Jones D., Chapman G.J., Siddle H.J., Russell D.A., Alazmani A., Culmer P. (2020). A Review of Wearable Sensor Systems to Monitor Plantar Loading in the Assessment of Diabetic Foot Ulcers. IEEE Trans.-Bio-Med. Eng..

[33] Castro-Martins P., Marques A., Coelho L., Vaz M., Baptista J.S. (2024). In-Shoe Plantar Pressure Measurement Technologies for the Diabetic Foot: A Systematic Review. Heliyon.

[34] Du L., Zhu X., Zhe J. (2015). An Inductive Sensor for Real-Time Measurement of Plantar Normal and Shear Forces Distribution. IEEE Trans. Biomed. Eng..

[35] Wang L., Jones D., Chapman G.J., Siddle H.J., Russell D.A., Alazmani A., Culmer P. Design of a Digital Triaxial Force Sensor for Plantar Load Measurements. Proceedings of the 2019 IEEE Sensors.

[36] Lord M., Hosein R. (2000). A Study of In-Shoe Plantar Shear in Patients with Diabetic Neuropathy. Clin. Biomech..

[37] Mori T., Hamatani M., Noguchi H., Oe M., Sanada H. (2012). Insole-Type Simultaneous Measurement System of Plantar Pressure and Shear Force During Gait for Diabetic Patients. J. Robot. Mechatronics.

[38] Tang J., Bader D.L., Moser D., Parker D.J., Forghany S., Nester C.J., Jiang L. (2023). A Wearable Insole System to Measure Plantar Pressure and Shear for People with Diabetes. Sensors.

[39] Amemiya A., Noguchi H., Oe M., Takehara K., Ohashi Y., Suzuki R., Yamauchi T., Kadowaki T., Sanada H., Mori T. (2020). Factors Associated with Callus Formation in the Plantar Region through Gait Measurement in Patients with Diabetic Neuropathy: An Observational Case-Control Study. Sensors.

[40] Crossland S.R., Siddle H.J., Brockett C.L., Culmer P. (2023). Evaluating the Use of a Novel Low-Cost Measurement Insole to Characterise Plantar Foot Strain during Gait Loading Regimes. Front. Bioeng. Biotechnol..

[41] Wang H., De Boer G., Kow J., Alazmani A., Ghajari M., Hewson R., Culmer P. (2016). Design Methodology for Magnetic Field-Based Soft Tri-Axis Tactile Sensors. Sensors.

[42] Wang H., Jones D., de Boer G., Kow J., Beccai L., Alazmani A., Culmer P. (2018). Design and Characterization of Tri-Axis Soft Inductive Tactile Sensors. IEEE Sens. J..

[43] Rajala S.N.K., Lekkala J. (2014). Plantar Shear Stress Measurements—A Review. Clin. Biomech..

[44] Viry L., Levi A., Totaro M., Mondini A., Mattoli V., Mazzolai B., Beccai L. (2014). Flexible Three-Axial Force Sensor for Soft and Highly Sensitive Artificial Touch. Adv. Mater..

[45] Lee H.K., Chung J., Chang S.I., Yoon E. (2011). Real-Time Measurement of the Three-Axis Contact Force Distribution Using a Flexible Capacitive Polymer Tactile Sensor. J. Micromech. Microeng..

[46] Santos V.M., Gomes B.B., Neto M.A., Amaro A.M. (2024). A Systematic Review of Insole Sensor Technology: Recent Studies and Future Directions. Appl. Sci..

[47] Bahin L., Tourlonias M., Bueno M.A., Sharma K., Rossi R.M. (2023). Smart Textiles with Polymer Optical Fibre Implementation for In-Situ Measurements of Compression and Bending. Sens. Actuators A Phys..

[48] Zhou Y., Zhu W., Zhang L., Gong J., Zhao D., Liu M., Lin L., Meng Q., Thompson R., Sun Y. (2019). Magnetic Properties of Smart Textile Fabrics through a Coating Method with NdFeB Flake-like Microparticles. J. Eng. Fibers Fabr..

[49] Lewis E. (2023). Radiant Textiles: Designing Electromagnetic Textile Systems. University of Borås Studies in Artistic Research. Doctoral Thesis.

[50] Yang P.K., Lin L., Yi F., Li X., Pradel K.C., Zi Y., Wu C.I., He J.H., Zhang Y., Wang Z.L. (2015). A Flexible, Stretchable and Shape-Adaptive Approach for Versatile Energy Conversion and Self-Powered Biomedical Monitoring. Adv. Mater..

[51] Xu H., Tao J., Liu Y., Mo Y., Bao R., Pan C. (2022). Fully Fibrous Large-Area Tailorable Triboelectric Nanogenerator Based on Solution Blow Spinning Technology for Energy Harvesting and Self-Powered Sensing. Small.

[52] Fan W., He Q., Meng K., Tan X., Zhou Z., Zhang G., Yang J., Wang Z.L. (2020). Machine-Knitted Washable Sensor Array Textile for Precise Epidermal Physiological Signal Monitoring. Sci. Adv..

[53] Mokhtari F., Cheng Z., Raad R., Xi J., Foroughi J. (2020). Piezofibers to Smart Textiles: A Review on Recent Advances and Future Outlook for Wearable Technology. J. Mater. Chem..

[54] Zhang W., Diao D., Sun K., Fan X., Wang P. (2018). Study on Friction-Electrification Coupling in Sliding-Mode Triboelectric Nanogenerator. Nano Energy.

[55] Hudec R., Matúška S., Kamencay P., Benco M. (2021). A Smart IoT System for Detecting the Position of a Lying Person Using a Novel Textile Pressure Sensor. Sensors.

[56] Gleskova H., Ishaku A., Bednar T., Hudec R. (2022). Optimization of All-Textile Capacitive Sensor Array for Smart Chair. IEEE Access.

[57] Fobelets K., Thielemans K., Mathivanan A., Papavassiliou C. (2019). Characterization of Knitted Coils for E-Textiles. IEEE Sens. J..

[58] Tajitsu Y. (2020). Development of E-Textile Sewn Together with Embroidered Fabric Having Motion-Sensing Function Using Piezoelectric Braided Cord for Embroidery. IEEE Trans. Dielectr. Electr. Insul..

[59] Tajitsu Y., Takarada J., Hikichi T., Sugii R., Takatani K., Yanagimoto H., Nakanishi R., Shiomi S., Kitamoto D., Nakiri T. (2023). Application of Piezoelectric PLLA Braided Cord as Wearable Sensor to Realize Monitoring System for Indoor Dogs with Less Physical or Mental Stress. Micromachines.

[60] Uno M.O., Morita S., Omori M., Yoshimura K. (2022). Pressure Sensor Yarns with a Sheath-Core Structure Using Multi-Fiber Polymer. Sens. Actuators A Phys..

[61] Zhao J., Fu Y., Xiao Y., Dong Y., Wang X., Lin L. (2020). A Naturally Integrated Smart Textile for Wearable Electronics Applications. Adv. Mater. Technol..

[62] Souri H., Bhattacharyya D. (2018). Wearable Strain Sensors Based on Electrically Conductive Natural Fiber Yarns. Mater. Des..

[63] Lian Y., Yu H., Wang M., Yang X., Zhang H. (2020). Ultrasensitive Wearable Pressure Sensors Based on Silver Nanowire-Coated Fabrics. Nanoscale Res. Lett..

[64] Seyedin S., Razal J.M., Innis P.C., Jeiranikhameneh A., Beirne S., Wallace G.G. (2015). Knitted Strain Sensor Textiles of Highly Conductive All-Polymeric Fibers. ACS Appl. Mater. Interfaces.

[65] Santoni SPA (2025). Sangiacomo Star-D: Sangiacomo Electronic Knitting Machine. https://www.santoni.com/uploads/2023-10-16/STAR-D_E.pdf.

[66] Zhu Y., Assadian M., Ramezani M., Aw K.C. (2018). Printing of Soft Stretch Sensor from Carbon Black Composites. Proceedings.

[67] Honnet C., Perner-Wilson H., Teyssier M., Fruchard B., Steimle J., Baptista A.C., Strohmeier P. (2020). PolySense: Augmenting Textiles with Electrical Functionality Using In-Situ Polymerization. Conf. Hum. Factors Comput.-Syst. Proc..

[68] Souri H., Bhattacharyya D. (2019). Highly Stretchable and Wearable Strain Sensors Using Conductive Wool Yarns with Controllable Sensitivity. Sens. Actuators A Phys..

[69] Anike J.C., Le H.H., Brodeur G.E., Kadavan M.M., Abot J.L. (2017). Piezoresistive Response of Integrated CNT Yarns under Compression and Tension: The Effect of Lateral Constraint. C.

[70] Wiranata A., Ohsugi Y., Minaminosono A., Mao Z., Kurata H., Hosoya N., Maeda S. (2021). A DIY Fabrication Approach of Stretchable Sensors Using Carbon Nano Tube Powder for Wearable Device. Front. Robot..

[71] Dong T., Gu Y., Liu T., Pecht M. (2021). Resistive and Capacitive Strain Sensors Based on Customized Compliant Electrode: Comparison and Their Wearable Applications. Sens. Actuators A Phys..

[72] Xu P., Wang S., Lin A., Min H.K., Zhou Z., Dou W., Sun Y., Huang X., Tran H., Liu X. (2023). Conductive and Elastic Bottlebrush Elastomers for Ultrasoft Electronics. Nat. Commun..

[73] Muth J.T., Vogt D.M., Truby R.L., Mengüç Y., Kolesky D.B., Wood R.J., Lewis J.A. (2014). Embedded 3D Printing of Strain Sensors within Highly Stretchable Elastomers. Adv. Mater..

[74] Shintake J., Piskarev Y., Jeong S.H., Floreano D. (2018). Ultrastretchable Strain Sensors Using Carbon Black-Filled Elastomer Composites and Comparison of Capacitive Versus Resistive Sensors. Adv. Mater. Technol..

[75] Cholleti E.R., Stringer J., Assadian M., Battmann V., Bowen C., Aw K. (2019). Highly Stretchable Capacitive Sensor with Printed Carbon Black Electrodes on Barium Titanate Elastomer Composite. Sensors.

[76] Song P., Wang G., Zhang Y. (2021). Preparation and Performance of Graphene/Carbon Black Silicone Rubber Composites Used for Highly Sensitive and Flexible Strain Sensors. Sens. Actuators A Phys..

[77] Giffney T., Bejanin E., Kurian A.S., Travas-Sejdic J., Aw K. (2017). Highly Stretchable Printed Strain Sensors Using Multi-Walled Carbon Nanotube/Silicone Rubber Composites. Sens. Actuators A Phys..

[78] Matsuhisa N., Kaltenbrunner M., Yokota T., Jinno H., Kuribara K., Sekitani T., Someya T. (2015). Printable Elastic Conductors with a High Conductivity for Electronic Textile Applications. Nat. Commun..

[79] Matsuhisa N., Inoue D., Zalar P., Jin H., Matsuba Y., Itoh A., Yokota T., Hashizume D., Someya T. (2017). Printable Elastic Conductors by in Situ Formation of Silver Nanoparticles from Silver Flakes. Nat. Mater..

[80] Yoon I.S., Kim S.H., Oh Y., Ju B.K., Hong J.M. (2020). Ag Flake/Silicone Rubber Composite with High Stability and Stretching Speed Insensitive Resistance via Conductive Bridge Formation. Sci. Rep..

[81] Lu D., Wong C.P. (2000). Thermal Decomposition of Silver Flake Lubricants. J. Therm. Anal. Calorim..

[82] Zhang R., Lin W., Moon K.s., Wong C.P. (2010). Fast Preparation of Printable Highly Conductive Polymer Nanocomposites by Thermal Decomposition of Silver Carboxylate and Sintering of Silver Nanoparticles. ACS Appl. Mater. Interfaces.

[83] Clement J.L., Jarrett P.S. (1994). Antibacterial Silver. Met.-Based Drugs.

[84] DM-SAS-10010 Product Datasheet. https://www.dycotecmaterials.com/wp-content/uploads/2018/01/Dycotec-TDS_DM-SAS-10010-Datasheet-13.05.20.pdf.

[85] (2018). Plastics—Determination of Tensile Properties—Part 3: Test Conditions for Films and Sheets.

[86] Shen H., Ke H., Feng J., Jiang C., Wei Q., Wang Q. (2021). Highly Sensitive and Stretchable C-MWCNTs/PPy Embedded Multidirectional Strain Sensor Based on Double Elastic Fabric for Human Motion Detection. Nanomaterials.

[87] Li Y., Miao X., Chen J.Y., Jiang G., Liu Q. (2021). Sensing Performance of Knitted Strain Sensor on Two-Dimensional and Three-Dimensional Surfaces. Mater. Des..

